# Clinical and Echocardiographic Parameters Predicting 1- and 2-Year Mortality After Transcatheter Aortic Valve Implantation

**DOI:** 10.3389/fcvm.2021.739710

**Published:** 2021-12-06

**Authors:** Didrik Kjønås, Henrik Schirmer, Svend Aakhus, Jo Eidet, Siri Malm, Lars Aaberge, Rolf Busund, Assami Rösner

**Affiliations:** ^1^Department of Cardiology, University Hospital of North Norway, Tromsø, Norway; ^2^Department of Cardiology, Akershus University Hospital, Lørenskog, Norway; ^3^Institute of Clinical Medicine, University of Oslo, Oslo, Norway; ^4^Department of Circulation and Imaging, Faculty of Medicine and Health Science, Norwegian University of Science and Technology, Norwegian University of Science and Technology (NTNU), Trondheim, Norway; ^5^Clinic of Cardiology, St. Olavs University Hospital, Trondheim, Norway; ^6^Department of Anesthesiology, Oslo University Hospital Rikshospitalet, Oslo, Norway; ^7^Department of Cardiology, University Hospital of North Norway, Harstad, Norway; ^8^Department of Cardiology, Oslo University Hospital, Rikshospitalet, Oslo, Norway; ^9^Institute of Clinical Medicine, The Arctic University of Norway (UiT), Tromsø, Norway; ^10^Department of Cardiothoracic and Vascular Surgery, University Hospital of North Norway, Tromsø, Norway

**Keywords:** TAVI, mortality, echocardiography, strain, risk assessment

## Abstract

**Background:** Transcatheter aortic valve implantation (TAVI) has become a standard treatment option for patients with symptomatic aortic stenosis. Elderly high-risk patients treated with TAVI have a high residual mortality due to preexisting comorbidities. Knowledge of factors predicting futility after TAVI is sparse and clinical tools to aid the preoperative evaluation are lacking. The aim of this study was to evaluate if echocardiographic measures, including speckle-tracking analysis, in addition to clinical parameters, could aid in the prediction of mortality beyond 30 days after TAVI.

**Methods:** This prospective observational cohort study included 227 patients treated with TAVI at the University Hospital of North Norway, Tromsø and Oslo University Hospital, Rikshospitalet from February 2010 to June 2013. All the patients underwent preoperative echocardiographic evaluation with retrospective speckle-tracking analysis. Primary endpoints were 1- and 2-year mortality beyond 30 days after TAVI.

**Results:** All-cause 1- and 2-year mortality beyond 30 days after TAVI was 12.1 and 19.5%, respectively. Predictors of 1-year mortality beyond 30 days were body mass index [hazard ratio (HR): 0.88, 95% CI: 0.80–0.98, *p* = 0.018], previous myocardial infarction (HR: 2.69, 95% CI: 1.14–6.32, *p* = 0.023), and systolic pulmonary artery pressure ≥ 60 mm Hg (HR: 5.93, 95% CI: 1.67–21.1, *p* = 0.006). Moderate-to-severe mitral regurgitation (HR: 2.93, 95% CI: 1.53–5.63, *p* = 0.001), estimated glomerular filtration rate (HR: 0.98, 95% CI: 0.96–0.99, *p* = 0.002), and chronic obstructive pulmonary disease (HR: 1.9, 95% CI: 1.01–3.58, *p* = 0.046) were predictors of 2-year mortality.

**Conclusion:** Both the clinical and echocardiographic parameters should be considered when evaluating high-risk patients for TAVI, as both are predictive of 1-and 2-year mortality. Our results support the importance of individual risk assessment using a multidisciplinary, multimodal, and individual approach.

## Introduction

Transcatheter aortic valve implantation (TAVI) for the treatment of symptomatic aortic stenosis (AS) was initially reserved for patients with high or prohibitive risk for surgical aortic valve replacement (SAVR) (1, 2). Although there has been an expansion of indication for TAVI, uncertainties still exist with respect to criteria when TAVI is futile in terms of survival (3–5). Clinical tools for identification of these patients are lacking and surgical risk scores have shown limited applicability in this patient group (5–7). TAVI in elderly high-risk patients with symptomatic AS has shown to improve symptoms and prolong life compared to medical therapy (1, 2). However, these patients have a high residual mortality as a result of preexisting comorbidities, both the cardiac and non-cardiac in origin (8, 9). Identification of factors that predict survival after TAVI is important in order to improve patient selection and form a better foundation for informed consent. The evaluation of these patients is complex and requires a multidisciplinary approach and individualized risk assessment to identify, if any of these risk factors are modifiable pre-TAVI. The aim of this study was to investigate, if clinical parameters, in addition to preoperative echocardiographic evaluation, including conventional and speckle-tracking analysis of the right and left ventricle, could aid in the identification of patients with increased risk of mortality beyond the perioperative period.

## Materials and Methods

### Study Population

This study included 227 patients with severe symptomatic AS treated with TAVI at the University Hospital of North Norway, Tromsø and Oslo University Hospital, Rikshospitalet from February 2010 to June 2013. The patients were recruited continuously from the population offered TAVI during this study period at both the centers. Suitability of the patient for TAVI was determined by a multidisciplinary heart team considering comorbid status and cognitive function in conjunction with technical feasibility. Patients unable to give informed consent, low motivation for treatment, and life expectancy of <12 months were not offered TAVI. The primary endpoints of this study were to identify risk factors for 1- and 2-year mortality beyond 30 days after TAVI. This study was approved by the Regional Ethics Committees for Medical Research Ethics, North and South East Norway. All the patients gave a written informed consent.

Demographics, clinical characteristics, and postoperative mortality and complications of the patient were obtained from the electronic records of the patient. All the patients were offered outpatient follow-up at 6 and 12 months after TAVI and mortality data were obtained from the electronic records of the patient linked to the National Mortality Registry. Complications were classified according to the Valve Academic Research Consortium (VARC)-2 criteria (10). Peripheral artery disease (PAD) was defined as claudication, previous amputation due to vascular insufficiency, previous reconstructive surgery or percutaneous intervention, abdominal aortic aneurism, and/or >50% stenosis in a peripheral artery diagnosed by CT or angiographic imaging. Chronic obstructive pulmonary disease (COPD) was classified according to the GOLD classification. Patients with COPD of unknown grade were classified as having grade 1. Chronic and paroxysmal atrial fibrillation/flutter was grouped as one variable. Previous cerebrovascular events comprised both the previous strokes and transient ischemic attacks. Poor mobility was defined as severe impairment of mobility secondary to musculoskeletal or neuromuscular dysfunction.

The specific predictors identified were assessed in a separate and more recent cohort consisting of 258 patients treated with TAVI at the University Hospital of North Norway, Tromsø from January 2017 to September 2019. A local data protection officer approved the validation of our original results.

### Echocardiography

Preoperative transthoracic echocardiogram (TTE) evaluation was performed in all the patients using either an iE33 (S5-1 probe, Philips Medical systems, Andover, Massachusetts, USA) or a Vivid E9 (GE Vingmed, Horten, Norway, UK) scanner with a 2.5–3.5 MHz transducer. In the left lateral decubitus position, two-dimensional grayscale images were obtained in the apical four-, two-, and three-chambers and parasternal short- and long-axis views. Simpson's biplane method was used for estimating left ventricular ejection fraction (LVEF) and left atrial volume at end-systole was obtained from the same views. LV longitudinal function was assessed by mitral annular plane systolic excursion (MAPSE) in the septal and lateral mitral rings in the apical four-chamber view or the mean-value of both (MAPSE average). Intraventricular septum thickness in diastole was derived from M-mode images in the parasternal long-axis view. Mitral flow E velocity, E/A ratio, E/e' ratio, and E deceleration time were used for the assessment of LV diastolic function. LV stroke volume index was calculated from the left ventricular outflow tract (LVOT) diameter and LVOT velocity time integral. The degree of AS was expressed by the mean and peak gradient and peak velocity of the Doppler flow across the aortic valve and the aortic valve area from the continuity equation and the indexed area. The degree of aortic regurgitation (AR) was estimated by the size of the regurgitation area by color Doppler, pressure half time, and diastolic velocities in descending aorta by Doppler-flow signal. The degree of mitral regurgitation (MR) was based on measurement and visual assessment of color Doppler images, vena contracta, and/or calculation of proximal isovelocity surface area. The presence of mitral stenosis was evaluated by measuring mean gradients over the mitral valve in addition to pressure half time and valve area.

Right ventricular (RV) function was evaluated in an adjusted four-chamber view at the largest transversal diameter of the RV. Systolic RV function was assessed by tricuspid annular peak systolic excursion (TAPSE) and tissue Doppler-derived peak tricuspid annular systolic velocity (TASV) in the basal RV free wall. RV fractional area change (RV FAC) was calculated from RV end-diastolic and end-systolic areas. Systolic pulmonary artery pressure (SPAP) was derived from continuous wave Doppler measurements of tricuspid regurgitation (TR) adding an estimate of right atrial pressure derived from respiratory variation of the diameter of the inferior vena cava. When TR gradient was not recorded, SPAP was considered being <30 mm Hg. Pulmonary arterial hypertension (PHT) was categorized into mild (<30 mm Hg), moderate (30–59 mm Hg), and severe (≥60 mm Hg).

### Strain Analysis

Left ventricular longitudinal (myocardial) strain was estimated by analysis of the LV in the apical four-, two-, and three-chamber views. In this study, LV global longitudinal strain (LVGLS) was defined as the average of three peak strain values of the three views. RV longitudinal strain (RVLS) was estimated by analysis of the lateral RV wall only in an apical four-chamber view. The time point of the aortic valve closure was measured in continuous Doppler registrations of the aortic flow. GLS values were extracted from strain curves by defining the systolic time interval between R wave and the time point of aortic valve closure. Strain curves with artifacts due to reverberation, air artifact, or insufficient tracking were discarded based on subjective visual assessment. In patients with atrial dysrhythmia, strain from three cycles, if available, was obtained and averaged. All the strain analyses were performed using speckle-tracking software VVI7 (Siemens, Mountain View, California, USA).

### Transcatheter Aortic Valve Implantation Procedure

All the procedures were performed in general anesthesia using a first-generation self-expanding Medtronic CoreValve (Medtronic Incorporation, Minneapolis, Minnesota, USA) or either first- or second-generation Edwards SAPIEN XT balloon-expandable valve (Edwards Lifesciences, Irvine, California, USA). Transfemoral (TF) access was the preferred modality. Transapical (TA) access was used in the presence of highly calcified and tortuous pelvic vessels given acceptable LV and respiratory function. In the presence of inaccessible peripheral vessels and reduced LVEF or COPD, transaortic (TAo) access was used. Valve size was determined from the aortic annular diameter measured by CT scan reconstruction and/or transesophageal echocardiography.

### Statistical Analysis

Data are presented as mean ± SD or number (%) as appropriate. The Pearson's chi-squared test for percentages or independent *t*-test for continuous variables was used for comparing variables between groups. The univariate Cox regression analysis was performed for 1- and 2-year mortality where *p* < 0.15 was considered as statistically significant. Variables that differed significantly between groups and/or with *p* < 0.15 in the univariate analysis were selected and tested for colinearity and correlation prior to the backward multivariate Cox regression analysis. The Lambda and Pearson's correlation coefficients were used to determine significant correlation between nominal and continuous variables, respectively. The receiver operating characteristic (ROC) analysis was performed for continuous variables to determine a cutoff value for continuous variables. No imputation for missing data was performed and multivariable analysis was done on all the available patients for each analysis. *p* < 0.05 in multivariable analysis was considered as statistically significant. The power calculation package in the STATA version 12 was used for estimating the power of the study. A minimum detectable hazard ratio (HR) of 1.25 for mortality for a 1-unit change for each echocardiographic variable with a power of 80% with a 5% probability of a false-negative result was estimated. All the statistical analyses were done using the SPSS version 24 (SPSS Incorporation, Chicago, Illinois, USA).

To determine the inter- and intraobserver variability of longitudinal strain measurements, recordings from 30 patients were selected at random and another experienced observer repeated the analysis. The main observer reanalyzed the same data after several months. The intraclass correlation coefficient was used to test inter- and intraobserver variability (11).

## Results

Demographics, clinical characteristics, and periprocedural results with respect to 1- and 2-year mortality of the patient are given in Table 1 and the echocardiographic parameters are shown in Table 2. A total of nine patients were excluded from the final analysis due to lack of available echocardiographic images in five cases, one case with AR and not AS, and three cases did not undergo TAVI. All-cause mortality at 1 and 2 years was 19.7% (*n* = 43) and 26.6% (*n* = 58), respectively, including 30-day mortality at 8.7% (*n* = 19). These 19 patients were excluded and the final analysis included 199 patients. There was no loss to follow-up with respect to primary endpoints.

**Table 1:** Demographic, clinical, and periprocedural characteristics stratified according to 1- and 2-year mortality.

**Variable**	**Survivors 1-year**	**Non-survivors 1-year**	***P*-value**	**Survivors 2-years**	**Non-survivors 2-years**	***P*-value**
	**(*n* = 175)**	**(*n* = 24)**		**(*n* = 160)**	**(*n* = 39)**	
Age, years	82 ± 7	81 ± 7	0.920	82 ± 7	82 ± 7	0.748
Female	79 (45)	10 (42)	0.748	76 (48)	13 (33)	0.111
BMI, kg/m^2^	27 ± 5	24 ± 6	0.034	27 ± 5	25 ± 5	0.085
STS score	5.5 ± 3.3	9.2 ± 6	<0.0001	5.4 ± 3.3	7.9 ± 5.2	<0.0001
Euroscore 2	8.8 ± 6.4	13.8 ± 11	0.001	8.9 ± 6.5	11.5 ± 9.6	0.047
*NYHA class*			0.260			0.861
II	25 (14)	4 (17)		24 (15)	5 (13)	
III	109 (62)	11 (46)		97 (61)	23 (59)	
IV	41 (23)	9 (38)		39 (24)	11 (28)	
Heart failure	75 (43)	9 (38)	0.618	69 (43)	15 (38)	0.597
Hypertension	117 (67)	16 (67)	0.985	107 (67)	26 (67)	0.980
Atrial dysrhythmia	73 (42)	16 (67)	0.021	63 (39)	26 (67)	0.002
Coronary artery disease	118 (64)	17 (71)	0.738	109 (68)	26 (67)	0.861
Previous myocardial infarction	62 (35)	14 (58)	0.030	57 (36)	19 (49)	0.131
Previous PCI	73 (42)	5 (21)	0.049	67 (42)	11 (28)	0.117
Previous cardiac surgery	81 (46)	12 (50)	0.732	76 (48)	17 (44)	0.661
LBBB	16 (9)	2 (8)	0.897	14 (9)	4 (10)	0.769
Peripheral artery disease	58 (33)	8 (33)	0.985	51 (32)	15 (38)	0.433
Cerebrovascular disease	43 (25)	9 (38)	0.176	38 (24)	14 (36)	0.122
Previous cerebrovascular event	38 (22)	8 (33)	0.205	33 (21)	13 (33)	0.091
Immunocompromised	20 (11)	4 (17)	0.460	17 (11)	7 (18)	0.208
Diabetes	47 (27)	12 (50)	0.020	43 (27)	16 (41)	0.083
COPD	57 (33)	13 (54)	0.038	50 (31)	20 (51)	0.019
eGFR, ml/min/1.73 m^2^	60 ± 19	50 ± 24	0.011	61 ± 18	51 ± 23	0.004
Poor mobility	19 (11)	4 (17)	0.404	18 (11)	5 (13)	0.783
Access			0.700			0.369
Transfemoral	102 (58)	15 (63)		96 (60)	21 (54)	
Transaortic	23 (13)	4 (16)		19 (12)	8 (21)	
Transapical	50 (29)	5 (21)		45 (28)	10 (25)	
**Valve type**			0.453			0.206
Edwards Sapien	136 (78)	17 (71)		126 (79)	27 (69)	
CoreValve	39 (22)	7 (29)		34 (21)	12 (31)	
**Complications**						
Stroke	4 (2.3)	0 (0)	0.454	3 (2)	1 (2)	0.783
New permanent pacemaker^*^	14 (9)	3 (15)	0.415	13 (9)	4 (12)	0.641
Moderate to severe PVL	17 (10)	1 (4)	0.374	15 (9)	3 (8)	0.743
Vascular complications^**^	10 (6)	5 (21)	0.009	9 (6)	6 (15)	0.038
Major or life threatening bleeding	3 (2)	2 (8)	0.052	2 (1)	3 (8)	0.021
Myocardial infarction	3 (2)	0 (0)	0.518	2 (1)	1 (3)	0.546
Acute kidney injury	4 (2)	0 (0)	0.454	4 (3)	0 (0)	0.319
Sepsis	5 (3)	0 (0)	0.402	5 (3)	0 (0)	0.264
Other TAVI complications	10 (6)	3 (13)	0.207	8 (5)	5 (13)	0.076

*Values are mean ± SD or n (%)*.

*BMI, body mass index; COPD, chronic obstructive pulmonary disease; eGFR, estimated glomerular filtration rate; LBBB, left bundle branch block; NYHA, New York Heart Association; PCI, percutaneous coronary intervention; PVL, paravalvular leakage; STS, Society of Thoracic Surgeons; TAVI, transcatheter aortic valve implantation*.

* *171 patients did not have a pacemaker prior to TAVI*.

** *Includes minor and major vascular complications*.

**Table 2:** Preoperative echocardiographic parameter stratified according to 1- and 2-year mortality.

Variable^*^	**Survivors 1-year**	**Non-survivors 1-year**	***P*-value**	**Survivors 2-year**	**Non-survivors 2-years**	***P*-value**
	**(*n* = 175)**	**(*n* = 24)**		**(*n* = 160)**	**(*n* = 39)**	
LVEF, % (*n* = 194)			0.375			0.230
≥50	90 (53)	10 (42)		83 (54)	17 (43)	
31–49	60 (35)	12 (50)		53 (34)	19 (49)	
≤ 30	20 (12)	2 (8)		19 (12)	3 (8)	
LVGLS (*n* = 180)	−11.1 ± 3.8	−11.1 ± 4.1	0.991	−11.1 ± 3.8	−11.4 ± 4.0	0.645
MAPSE septal, cm (*n* = 193)	0.7 ± 0.3	0.6 ± 0.2	0.168	0.7 ± 0.3	0.6 ± 0.2	0.106
MAPSE lateral, cm (*n* = 193)	1.0 ± 0.3	0.9 ± 0.2	0.253	1.0 ± 0.3	0.9 ± 0.3	0.173
MAPSE average, cm (*n* = 193)	0.9 ± 0.3	0.8 ± 0.2	0.165	0.9 ± 0.3	0.8 ± 0.2	0.101
IVSDd, cm (*n* = 185)	1.2 ± 0.3	1.1 ± 0.3	0.403	1.2 ± 0.3	1.2 ± 0.3	0.592
AVA, cm^2^ (*n* = 196)	0.6 ± 0.2	0.6 ± 0.2	0.603	0.6 ± 0.2	0.6 ± 0.2	0.830
AVA index, cm^2^/m^2^ (*n* = 196)	0.3 ± 0.1	0.3 ± 0.1	0.857	0.3 ± 0.1	0.3 ± 0.1	0.534
AV gradient max, mmHg (*n* = 196)	84 ± 24	85 ± 31	0.798	85 ± 24	81 ± 27	0.410
AV gradient mean, mmHg (*n* = 197)	52 ± 15	51 ± 17	0.635	53 ± 16	49 ± 15	0.194
AV max velocity, m/s (*n* = 197)	4.5 ± 0.7	4.5 ± 0.8	0.710	4.6 ± 0.7	4.4 ± 0.7	0.237
LVOT diameter, cm	2.1 ± 0.3	2.1 ± 0.3	0.998	2.1 ± 0.2	2.1 ± 0.3	0.296
LVOT VTI, cm (*n* = 197)	19.8 ± 5.9	18.1 ± 5.9	0.181	19.8 ± 6.0	18.6 ± 4.9	0.230
SV index, ml/m^2^ (*n* = 197)	37 ± 11	36 ± 10	0.641	37 ± 11	37 ± 11	0.877
E/é (*n* = 135)	19.2 ± 8.3	18.9 ± 6.3	0.916	19 ± 8	19 ± 7	0.897
E/A (*n* = 137)	1.1 ± 0.6	1.2 ± 0.8	0.768	1.1 ± 0.6	1.3 ± 0.8	0.313
E deceleration time, ms (*n* = 197)	229 ± 92	214 ± 90	0.461	231 ± 94	212 ± 81	0.242
E velocity, cm/s (*n* = 197)	96 ± 34	95 ± 36	0.891	97 ± 34	91 ± 35	0.366
LA volume index, ml/m^2^ (*n* = 186)	53 ± 20	59 ± 20	0.192	52 ± 21	57 ± 19	0.213
MR moderate to severe (*n* = 194)	29 (17)	9 (38)	0.018	23 (15)	15 (38)	0.001
AR moderate to severe (*n* = 190)	27 (16)	8 (35)	0.031	25 (16)	10 (26)	0.160
Mitral stenosis	8 (5)	1 (4)	0.751	8 (5)	1 (2)	0.480
SPAP, mmHg			0.008			0.258
≥60	12 (7)	6 (25)		12 (7)	6 (15)	
30–59	104 (60)	14 (58)		95 (60)	23 (60)	
<30	59 (33)	4 (17)		53 (33)	10 (25)	
TAPSE, cm (*n* = 186)	1.6 ± 0.5	1.5 ± 0.5	0.235	1.6 ± 0.5	1.5 ± 0.6	0.309
TASV, cm/s (*n* = 120)	9.6 ± 3.2	8.8 ± 2.8	0.455	9.7 ± 3.3	8.5 ± 2.6	0.169
RV FAC, % (*n* = 179)	36 ± 13	35 ± 12	0.820	36 ± 13	35 ± 12	0.734
RVEDA, cm^2^ (*n* = 179)	19 ± 5	22 ± 5	0.029	20 ± 5	21 ± 5	0.155
RVESA, cm^2^ (*n* = 179)	13 ± 5	14 ± 4	0.107	13 ± 5	14 ± 4	0.267
TR moderate to severe (*n* = 194)	29 (17)	11 (46)	0.001	26 (17)	14 (36)	0.008
RVLS, % (*n* = 154)	−16.4 ± 6.5	−17.1 ± 7.2	0.667	−17 ± 7	−16 ± 7	0.875

*Values are mean ± SD or n (%)*.

*AR, aortic regurgitation; AV, aortic valve; AVA, AV area; FAC, fractional area change; IVSDd, intraventricular septum diameter in diastole; LA, left atrium; LVGLS, left ventricular global longitudinal strain; LVEF, left ventricular ejection fraction; LVOT, left ventricular outflow tract; MAPSE, mitral annular plane systolic excursion; MR, mitral regurgitation; MV, mitral valve; RVEDA, right ventricular end-diastolic area; RVESA, right ventricular end-systolic area; RVLS, right ventricular longitudinal strain; SPAP, systolic pulmonary artery pressure; SV, stroke volume; TAPSE, tricuspid annular peak systolic excursion; TASV, tricuspid annular systolic velocity; TR, tricuspid regurgitation*.

* *Numbers in brackets indicate the number of cases where the measurement was available*.

Of all the 30-day survivors after TAVI, 1-year mortality was 12.1% (*n* = 24) and 2-year mortality was 19.5% (*n* = 39). As shown in Table 1, these patients had a higher burden of comorbidities with higher risk estimated by conventional surgical risk scores. Atrial fibrillation/flutter, COPD, and reduced estimated glomerular filtration rate (eGFR) were significantly more prevalent in both the mortality groups. The only echocardiographic measures significantly more frequent in both the mortality groups were moderate-to-severe MR and TR. The incidence of major bleeding perioperatively and vascular complications was also higher in the mortality groups, although few in number.

Those who died within 1 year had significantly lower body mass index (BMI), higher incidence of diabetes and previous myocardial infarction (MI), and fewer patients had undergone percutaneous coronary intervention (PCI). They also had a significantly higher incidence of SPAP ≥ 60 mm Hg and moderate-to-severe AR pre-TAVI in addition to higher RV end-diastolic area (RVEDA). There was significant positive correlation between higher SPAP and higher RVEDA (*p* < 0.000), but no difference in stroke volume index (SVI). Lower BMI, previous cerebrovascular events, and diabetes were borderline significant between the 2-year mortality group and survivors.

**Figure 1:**
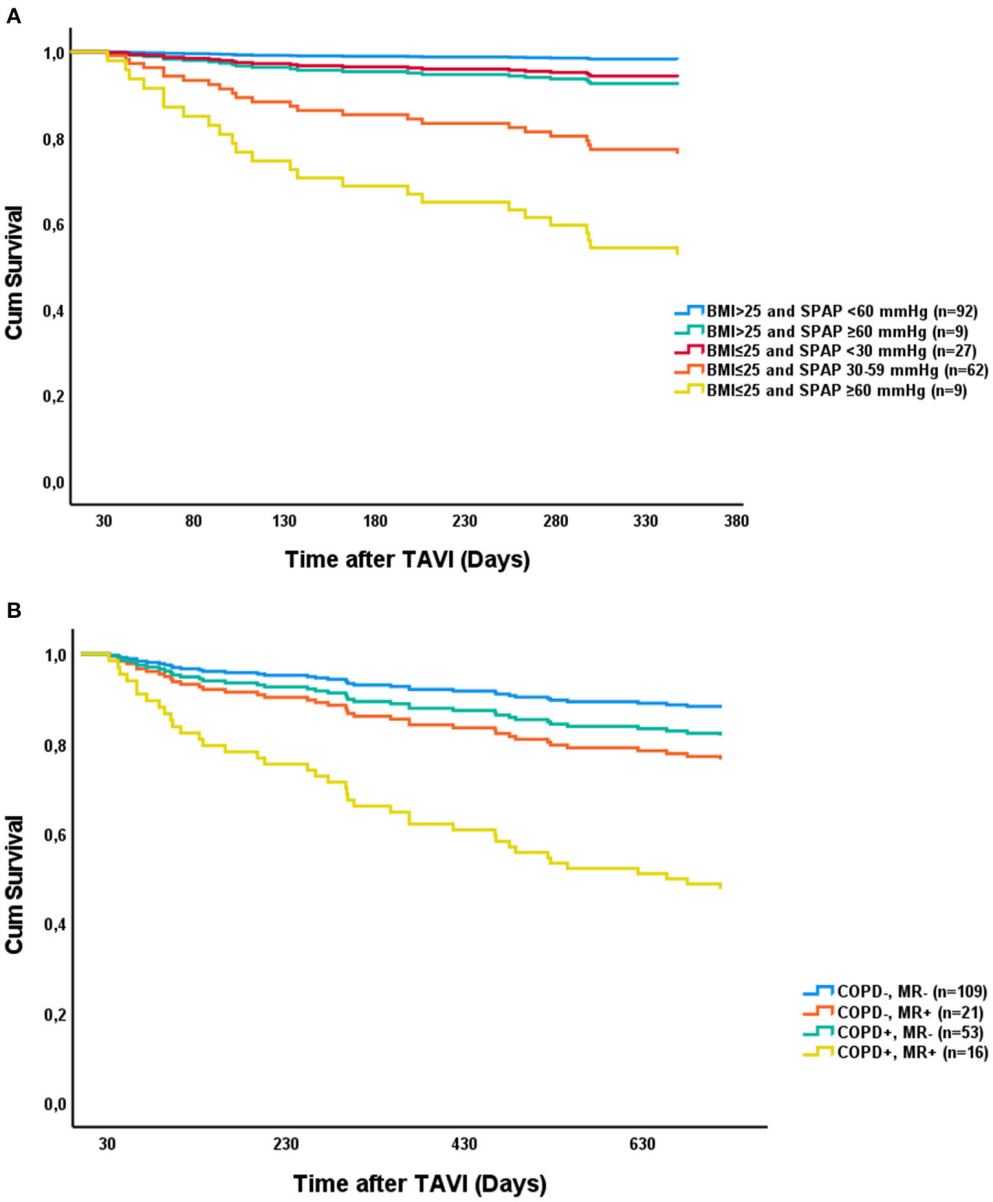
Survival curves representing the Cox proportional hazards analysis for: BMI (kg/m^2^) and SPAP grouped and adjusted for previous MI **(A)** with respect to 1-year mortality and moderate-to-severe MR and COPD grouped adjusted for eGFR (ml/min/1.73 m^2^) with respect to 2-year mortality **(B)**. BMI, body mass index; COPD, chronic obstructive pulmonary disease; eGFR, estimated glomerular filtration rate; MI, myocardial infarction; MR, mitral regurgitation; SPAP, systolic pulmonary artery pressure.

The results of the univariate and the multivariate Cox regression analysis are shown in Table 3. We identified lower BMI, previous MI, and SPAP ≥ 60 mm Hg as independent predictors of all-cause 1-year mortality beyond 30 days. Although not significant in multivariable analysis, there was a trend toward increased mortality in patients with SPAP 30–59 mm Hg. ROC analysis identified a cutoff value for BMI of 25 kg/m^2^ with a sensitivity of 0.8 and 1 specificity of 0.4. All the patients with SPAP ≥ 60 mm Hg in the mortality group died within the first year after TAVI. Thus, all the survivors with SPAP ≥ 60 mm Hg alive after 1 year were alive at 2 years. Figure 1A shows the survival curves for BMI and SPAP adjusted for previous MI. Of the patients with BMI > 25 kg/m^2^ and SPAP < or ≥ 60 mm Hg, 98 and 89% patients were alive after 1 year, respectively. In patients with BMI ≤ 25 kg/m^2^ and SPAP <30 mm Hg, SPAP 30–59 mm Hg, or SPAP ≥ 60 mm Hg, 1-year survival was 93, 77, and 44%, respectively. In patients with BMI ≤ 25 kg/m^2^ and SPAP ≥ 30 mm Hg, the survival at 1 year for patients without previous MI was 88% compared to 55% in patients with previous MI (*p* = 0.030). Predictors of all-cause 2-year mortality were moderate-to-severe MR, COPD, and reduced eGFR. Previous cerebrovascular event was borderline significant (*p* = 0.078). Figure 1B shows COPD and moderate-to-severe MR grouped adjusted for eGFR. The main impact on survival is the presence of both the COPD and moderate-to-severe MR. In patients without COPD and moderate-to-severe MR, 87% patients were alive after 2 years compared to only 44% patients in the presence of both the conditions.

**Table 3:** Results of the univariate and multivariate regression analysis of mortality at 1 and 2 years beyond 30 days after TAVI.

**Variable**	**1-year mortality**						**2-year mortality**					
	**Univariable**			**Multivariable**			**Univariable**			**Multivariable**		
	**HR**	**95% CI**	***P* value**	**HR**	**95% CI**	***P* value**	**HR**	**95% CI**	***P* value**	**HR**	**95% CI**	***P* value**
Atrial dysrhythmia	2.60	1.11–6.07	0.027	–	–	NS	2.70	1.39–2.52	0.003	–	–	NS
BMI, kg/m^2^	0.90	0.81–0.99	0.028	0.88	0.80–0.98	0.018	0.94	0.87–1.00	0.07	–	–	NS
COPD	2.39	1.03–5.11	0.043	–	–	NS	2.09	1.12–3.92	0.021	1.9	1.01–3.58	0.046
Diabetes	2.61	1.17–5.81	0.019	–	–	NS	1.83	0.97–3.46	0.064	–	–	NS
eGFR, ml/min/1.73 m^2^	0.97	0.95–0.99	0.009	–	–	NS	0.98	0.96–0.99	0.002	0.98	0.96–0.99	0.002
Previous MI	2.41	1.07–5.43	0.034	2.69	1.14–6.32	0.023	1.70	0.89–3.13	0.11	–	–	NS
Previous PCI	0.39	0.15–1.04	0.061	–	–	NS	0.57	0.28–1.14	0.113	–	–	NS
Previous CVE	–	–	NS	–	–	NS	1.78	0.91–3.46	0.09	–	–	NS
AR moderate to severe	2.58	1.09–6.09	0.03	–	–	NS	1.78	0.86–3.66	0.12	–	–	NS
MR moderate to severe	2.55	1.12–5.84	0.26	–	–	NS	2.87	1.51–5.48	0.001	2.93	1.53–5.63	0.001
SPAP, mmHg												
<30	–	–	–	–	–	–	–	–	–	–	–	–
30–59	1.91	0.63–5.80	0.254	2.10	0.66–6.45	0.21	1.26	0.60–2.65	0.54	–	–	NS
≥60	6.32	1.79–22.5	0.004	5.93	1.67–21.1	0.006	2.62	0.95–7.23	0.062	–	–	NS

*Data are displayed as hazard ratio (HR), 95% CI, with corresponding p-value*.

*NS, not significant; TAVI, transcatheter aortic valve implantation; AR, aortic regurgitation; BMI, body mass index; COPD, chronic obstructive pulmonary disease; CVE, cerebrovascular event; eGFR, estimated glomerular filtration rate; MI, myocardial infarction; MR, mitral regurgitation; PCI, percutaneous coronary intervention; SPAP, systolic pulmonary artery pressure*.

Strain analysis did not provide additional predictive value and remained statistically insignificant when evaluated in EF subgroups. As previously reported, the intraclass correlation coefficient for longitudinal strain measurement was 0.799 (95% CI: 0.695–0.868) and 0.924 (95% CI: 0.885–0.950) for inter- and intraobserver variability, respectively (11).

In the more recent validation cohort, 30-day mortality was 1.6% (*n* = 4). There was no significant difference in mortality beyond 30 days after TAVI between the original study cohort and the validation cohort after 1 and 2 years (12.1 vs. 9.1%, *p* = 0.30 and 19.5 vs. 16.1%, *p* = 0.34, respectively). The validation cohort had significantly lower incidence of previous MI (23 vs. 38%, *p* > 0.001) and moderate-to-severe MR (19.1 vs. 9.4%, *p* = 0.002), in addition to better eGFR (64 vs. 59 ml/min/1.73 m^2^, *p* = 0.006). There was no significant difference in the incidence of COPD (*p* = 0.273), SPAP ≥ 60 mm Hg (*p* = 0.489), or BMI (*p* = 0.291) between the two cohorts. Based on the factors identified and the Cox proportional hazards model from the original cohort, reduced eGFR remained the only significant predictor of mortality at 2 years after multivariable adjustment (HR: 0.98, 95% CI: 0.96–0.99, *p* < 0.001) in the more recent and less comorbid cohort.

## Discussion

Based on our original study cohort of high-risk patients, we found 1-year mortality 30 days beyond TAVI to be predicted by low BMI, increased SPAP, and a history of previous MI. COPD, moderate-to-severe MR, and reduced eGFR were predictors of mortality at 2 years. In this study, all-cause mortality at 1- and 2 years is similar to international registry data from the same period (12, 13). We did not include 30-day mortality in an attempt to better identify risk factors not influenced by perioperative factors. Predictors of mortality identified in this study, both the clinical parameters and echocardiographic measures, have previously been described in registry studies (9, 12–15). This study evaluated more parameters than those included in registry studies indicating the possible importance of the factors identified in this patient population. Longitudinal strain analysis of the left and right ventricle, in addition to a thorough preoperative echocardiographic evaluation, did not yield any additional predictive value. Besides reduced eGFR, the factors identified were not significant when evaluated in a more recent and less comorbid cohort with similar 1- and 2-year mortality rates 30 days beyond TAVI. However, our results are likely still relevant for a subgroup of high-risk patients in the current TAVI population.

In this study, the only independent echocardiographic predictors of mortality beyond 30 days were SPAP ≥ 60 mm Hg and moderate-to-severe MR for 1- and 2-year mortality, respectively. Neither EF nor longitudinal function, including longitudinal strain, differed between groups. PHT has been shown to predict poor outcome after TAVI (15, 16). O'Sullivan et al. found that precapillary PHT and combined pre- and postcapillary PHT were predictive of 1-year mortality; however, isolated postcapillary (LV induced) PHT was not predictive of 1-year mortality (17). Postcapillary PHT is a possible reversible condition improving after treatment of AS and concomitant heart failure. Thus, in patients with severe PHT, a thorough evaluation of its underlying cause and possible reversibility pre-TAVI are necessary for individualized risk assessment. Both the mortality groups had more than twice the prevalence of moderate-to-severe MR and moderate-to-severe MR was an independent predictor of 2-year mortality. In a meta-analysis by Nombela-Franco et al., moderate-to-severe MR was associated with increased 1-year mortality despite an improvement in the severity of regurgitation in approximately 50% of patients post-TAVI (18). MR might be organic or functional, the latter being the most common and most likely to improve post-TAVI. Whether or not MR should be treated concomitant with AS that remains a topic for further study, it is not known if the treatment of MR before or after TAVI will reduce risk of long-term mortality (19). A thorough evaluation of its cause could be beneficial prior to TAVI and medical therapy should be optimized regardless of etiology.

Previous MI and the presence and complexity of coronary artery disease (CAD) have been associated with 1-year mortality (13, 20, 21). We found previous MI to be an independent predictor of 1-year mortality, but no difference in incidence of CAD between the survivors and the mortality group. Previous MI could result in ischemic heart failure and cause substrates for arrhythmias in patients with AS, which might explain the current finding. CAD is prevalent among patients with AS evaluated for TAVI, especially in the high-risk groups (1, 22). CAD is a heterogeneous condition frequently associated with other comorbidities and the extent of myocardium at risk differs. In elderly high-risk patients undergoing TAVI, complete revascularization is not necessarily a prerequisite for favorable outcome (23). Patients with CAD, with or without previous MI, evaluated for TAVI that could benefit from an individualized revascularization strategy based on the complexity and severity of CAD taking into consideration the extent of myocardium at risk.

Chronic obstructive pulmonary disease is known to have a negative impact on survival after TAVI (9, 24). In our cohort, there was a significant difference in the incidence of COPD between survivors and 1- and 2-year mortality, with COPD being an independent predictor of 2-year mortality. A study by Mok et al. showed that patients with COPD had a >1.5 risk of death at midterm follow-up after TAVI and most of these patients died of respiratory failure secondary to COPD (25). Despite being an irreversible condition with a poor prognosis, patients with COPD may benefit from more intensive follow-up during treatment for concomitant disease both prior to and beyond initial perioperative period. Various degrees of renal failure are common in patients treated with TAVI and its impact on poor outcome is well-documented (26). The cause is likely multifactorial including advanced age, association between renal failure and other comorbidities, and increased risk of periprocedural complications. Although depending on stage, renal failure needs to be taken into consideration in the preoperative evaluation.

Frailty measures have been associated with worse outcome following TAVI (27). A study by Martin et al. from the UK TAVI registry showed that frailty measures, including poor mobility, were predictive of 1-year mortality (28). In this study, the incidence of poor mobility did not differ between the groups, albeit further frailty measures were not performed. Frailty can be described as an age-related syndrome characterized by physiological decline and vulnerability to adverse health events. Although we did not use a specific score to evaluate degree of frailty in this study, patients considered too frail by the multidisciplinary team were not offered treatment. This included severe immobility and dementia. Compared to current clinical practice, the criteria for treatment in this study were strict. There is still a debate on how to best assess it due to instrument variability, but the most cited includes weight loss as one of its components (29, 30). A meta-analysis and systematic review by Lv et al. showed that a high BMI was associated with reduced short- and long-term mortality corresponding to our results (31). The reason for this apparent “obesity paradox” is not yet clear. Several mechanisms have been suggested including younger age, earlier diagnosis, higher metabolic reserve, and cardiac cachexia. Patients with low BMI and/or malnourishment might benefit from prehabilitation prior to treatment to counteract the apparent negative effects of lower BMI and other frailty measures.

Guidelines from both the European Society of Cardiology and the American Heart Association emphasize the importance of a multidisciplinary evaluation by a heart team prior to TAVI considering technical aspects, comorbid status, expected benefits, and the preferences of the patients (32, 33). In addition to technical feasibility, criteria for when TAVI is futile are now included in guidelines, but the decision to not offer interventional treatment is still often challenging. The heterogeneity of the current TAVI population with respect to comorbid profile and improved patient selection, in addition to continuously evolving valve and sheath technology, warrants further study of TAVI subgroups and their individual risk profiles.

### Study Limitations

This study has several limitations. First, this study included relatively few patients from only two centers and was performed during an early stage after the implementation of TAVI as a treatment option. Second, all the procedures were performed in general anesthesia. At present, the majority of patients are treated using TF access performed in local anesthesia. The negative impact of general anesthesia on outcome would likely be evident early post-TAVI. Since we only included patients who survived beyond 30 days, the use of general anesthesia does not probably affect our results nor make it less representable for the current subgroup of patients with high-risk TAVI. Third, we used only first- and second-generation valves, whereas third- and fourth-generation valves are currently in use. Complications related to valve deployment and vascular injury were associated with unfavorable perioperative outcome and were more frequent with early valve generations. Beyond the perioperative period evaluated in this study, paravalvular leak (PVL) is the isolated valve-related factor most strongly associated with mortality. Although less frequent, it is still an issue with new-generation valves. Despite relatively low incidence of PVL in this study with no difference in incidence between groups, valve generation must be taken into consideration when interpreting our results in light of continuous improvements in valve technology. Fourth, this study population was older with more comorbidities than the current TAVI population in general. All these factors might make our results less generalizable today. Lastly, this study had relatively low power and event rate and our results might, therefore, be over fitted. However, the factors identified were highly significant and are strengthened by similar findings in previous research. Despite the TAVI population now being generally less comorbid than in this study, our results are still relevant when evaluating high-risk patients in the current TAVI population.

## Conclusion

Despite expanding indications for TAVI, there is still an unmet need for better identification of patients where TAVI is futile. Prediction of futility of treatment in terms of mortality after TAVI in elderly high-risk patients is challenging and requires a multidisciplinary, multimodal, and individual approach to diagnosis, treatment, and follow-up. Factors identified as predictors of mid- and long-term outcome after TAVI vary between studies indicative of the heterogeneity of this patient population. Certain preexisting conditions have inherent poor prognosis, where some are irreversible and others are amendable to optimization both before and after treatment. Active treatment and close follow-up of patients with high comorbid burden before, during, and after TAVI might ameliorate the inherent risks of preexisting conditions.

## Data Availability Statement

The datasets presented in this article are not readily available because the data used in our study can unfortunately not be shared, even upon request. The reason being that it contains sensitive information that could compromise patient anonymity and contain sensitive medical information. The access and distribution of such data are strictly regulated by law. Requests to access the datasets should be directed to didrikkj@gmail.com.

## Ethics Statement

The studies involving human participants were reviewed and approved by Regional Ethical Committee for Medical Research Ethics North Norway and Regional Ethical Committee for Medical Research Ethics South East Norway. The patients/participants provided their written informed consent to participate in this study.

## Author Contributions

DK: methodology, validation, formal analysis, investigation, data curation, writing-original draft, and visualization. HS: conceptualization, methodology, formal analysis, and writing-original draft. SA: conceptualization, resources, data curation, and writing-review and editing. JE: investigation, resources, data curation, and writing-review and editing. SM and LA: investigation, resources, and writing-review and editing. RB: investigation, resources, data curation, and writing-original draft. AR: conceptualization, methodology, validation, investigation, resources, data curation, writing-original draft, visualization, supervision, project administration, and funding acquisition. All authors contributed to the article and approved the submitted version.

## Funding

This work was supported and funded by the Regional Health Authorities of North Norway. The Arctic University of Norway provided funds for open access publication fees.

## Conflict of Interest

RB is a proctor for Edwards Lifesciences and has received speakers fee from Abbot. LA is a proctor for Edwards Lifesciences. AR and DK received financial support from the Regional Health Authorities of North Norway. The remaining authors declare that the research was conducted in the absence of any commercial or financial relationships that could be construed as a potential conflict of interest.

## Publisher's Note

All claims expressed in this article are solely those of the authors and do not necessarily represent those of their affiliated organizations, or those of the publisher, the editors and the reviewers. Any product that may be evaluated in this article, or claim that may be made by its manufacturer, is not guaranteed or endorsed by the publisher.
